# Assessment of dietary habits, nutritional status and common health complications of older people living in rural areas of Bangladesh

**DOI:** 10.1016/j.heliyon.2022.e08947

**Published:** 2022-02-11

**Authors:** Arafat Hassan Razon, Md.Imamul Haque, Md.Foyaj Ahmed, Tanvir Ahmad

**Affiliations:** Department of Nutrition and Food Technology, Jashore University of Science and Technology, Bangladesh

**Keywords:** Malnutrition, Nutritional status, Dietary knowledge, Simplified Nutritional Appetite Questionnaire (SNAQ)

## Abstract

**Background:**

Old age is one of the vulnerable and prone stages in terms of health status. So this study aimed to assess the nutritional status and common health complications of older people.

**Methods:**

Simplified Nutritional Appetite Questionnaire (SNAQ), Anthropometric measurements, Diet History Method, and Mini Nutritional Assessment (MNA) tools were used to measure the nutritional status. Data were analyzed by using Statistical Package for Social Science (SPSS) version 16.

**Results:**

Out of the total 320 elderly participants the mean ± SD value for the age of male and female was 67.25 ± 6.5 and 67.32 ± 7.7 years respectively. According to BMI classification, it was noticed that with advancing age the percentage of underweight was also increased such as for 60–75 years old age group the underweight percentage was 30.0% where for 76 to 85 and >85 years old age group the underweight percentage was 45.0% and 60.0% respectively. According to the MNA score, 97 elderly respondents were malnourished and a total of 172 respondents had SNAQ scores below 14. This study found a statistically significant (P < 0.05) correlations among various health complications with nutritional status according to MNA score. In addition 56.6% (OR = 1.24, 95% CI = .799–1.939), 63.8% (OR = 1.18, 95% CI = .745–1.857) and 64.7% (OR = 1.14, 95% CI = .720–1.804) respondents had diabetes mellitus, hypertension and cardiovascular disease respectively. The risk of musculoskeletal pain (OR = 1.073, 95% CI = .684–1.681), bedsore (OR = 1.884, 95% CI = .903–3.934) and decreased sense of thirst (OR = 1.278, 95% CI = .821–1.991) were higher among females than males. A little number of the elderly used to take milk, meat, and fish daily.

**Conclusion:**

During this cross-sectional study, significant correlations among nutritional changes with health complications were determined. To prevent malnutrition among the elderly a proper health policy as well as periodical nutritional screening should be conducted.

## 1 Introduction

It is very important to maintain a healthy nutritional status at any age. As a result, geriatric nutrition is the nutrition that helps to minimize the effects of aging and diseases as well as it helps to manage the physical, psychological and psychosocial states of the elderly population [1]. The elderly population may be defined as those populations whose age is greater than 65 years of age. The elderly can be classified into two types such as early elderly (between 65 to 74 years of age) and late elderly (above 75 years of age) [2]. In developing countries, it is very important to conduct more research to assess the nutritional status of the aging population. To guide community awareness and interventions the international dietary guidelines for older populations are required [3]. It has been predicted that from 2000 to 2030 about 550–930 million of world population may have age above 60 years. So worldwide it will be increased from 6.9% to 12% but in Asia 6%–12% [4]. In Bangladesh people who has age 60 years and above is considered an elderly person [5]. The aging of the population is now a global issue and this is also an emerging issue in Bangladesh [6]. It was evident that in Bangladesh the number of older people aged 60 and above was around 9.41 million in 2007 and this increasing tendency of older people was started in Bangladesh from 1951 [7, 8]. Throughout the world, twenty countries have the highest elderly population and Bangladesh is one of them. It is predicted that Bangladesh will achieve 44% of the world's elderly population by 2025 [9]. Among the 160 million population of Bangladesh, about 7% of the population has aged over 60 years and it will rise to 16% by 2050. The female elderly population is more malnourished than the male elderly population in Asian countries. But as a whole, both groups are highly vulnerable to malnutrition [10]. Due to extreme poverty the majority of older people in Bangladesh are unable to meet their nutritional requirements and this is a common scenario of poor families living in both urban and rural areas [11, 12]. The majority of the older people in Bangladesh have very poor socio-economic conditions because of poverty, wage discrimination, want of essential goods and commodities, shelter, and compulsory retirement from the job when the age limit is attained [13]. The nutritional status and food intake pattern of the older people can be affected by various changes such as physical, psychological, social, or health-related changes. Despite these changes, proper nutrition and a healthy lifestyle can only improve the nutritional status and quality of life among the elderly population [14]. Several studies found some medical complications associated with older people. A study found that a huge number of older people had medical complications like cataracts, joint pain, hypertension, diabetes mellitus, etc [15]. To minimize the adverse nutritional outcome it is very important to conduct a nutritional assessment in older people to detect malnutrition. BMI is one of the most popular nutritional assessment tools and it is measured by using the weight and height of a person and is expressed as Kg/m2. It can be used in many nutritional screening programs and it is an appropriate identifier of malnutrition. A person having a BMI below 18.5 kg/m^2^ is considered undernourished or malnourished [16, 17]. Another simplified nutritional screening tool is Mid Upper Arm Circumference (MUAC) and which can be used as an identifier for the health and nutritional status of elderly individuals [18, 19, 20]. Among the various nutritional assessment tools "Mini Nutritional Assessment" (MNA) tool is one of them. The development of the MNA tool was started in 1989 in a meeting of the "International Association of geriatrics and Gerontology" (IAGG). Older people can be classified as nourished, at risk for malnutrition, or malnourished through the MNA tool. MNA is performed in a two-step process to assess the nutritional status. About 10–15 min are required to perform a complete MNA [21]. Another nutritional assessment tool is the "Simplified Nutritional Appetite Questionnaire” (SNAQ). It was invented in 2005 by the Council for Nutritional Strategies. It is usually used to predict weight loss which is a common condition and always a serious event in older people [22]. As older people are very much vulnerable to nutritional deficiency diseases so this study has utilized various nutritional assessment tools to assess the nutritional status and common health complications among the elderly population of a rural area in Jashore, Bangladesh.

## 2 Materials and methods

### 2.1 Study design

A cross-sectional study was conducted to describe the nutritional status and common health complications among elderly individuals (60 years old or older) living in rural areas of jashore city under randomly selected villages such as Islampur, Dogachia, Shamnagar, Belermath, Kamlapur, Jogahati, Saziali, Abdulpur, and Vogolpur.

### 2.2 Population

The study was carried out through the inclusion of individuals aged 60 years or older that lived in the above-mentioned villages. Moreover, 320 elderly individuals were included in this study.

### 2.3 Eligibility criterias

Those eligible to participate in the study were individuals of both sexes, aged ≥60 years living in rural areas under the above-mentioned villages of Jashore city who agreed to participate in the research. The exclusion criteria comprised: those who disagreed to give consent; geriatric individuals who were not willing to give an interview; seriously ill people and <60 years old people.

### 2.4 Data collection techniques

The study was conducted from September 2019 to February 2020 and data were collected from November 2019 to December 2019 through a simple random sampling method. The study was conducted using the direct survey method PAPI (Paper and Pen Personal Interview). The sample size of this study was calculated through the use of an equation [1] and that equation [1] was previously used in a similar study like this [4].

(1) N = Z^2^pq/d^2^

Here, Z = 1.96, p = 0.5 (as no study found), q = 0.5 and d = 0.05. According to this equation [1] the sample size was approximately 384 but due to lack of funding and time constrain only 320 samples were included in this study. After calculating the approximate sample size for this study the above-mentioned villages were selected purposively because these villages were situated near Jashore city and it was very easy as well as economical to reach these villages. Then through a simple random sampling method, the required data were collected from the older people of each village and these villages were visited according to their alphabetical order. As this study followed a simple random sampling method that's why data were collected randomly from each villages and at the end of the data collection period a total of 320 data were gathered from these villages.An instrument was developed by the researchers to collect the socio-economic, socio-demographic, anthropometric, and dietary history data. Weight was measured using a digital scale (Soehnle; CMS, London, UK) and height was measured using a stadiometer (Cranlea Ltd, Birmingham, UK). The questionnaire was prepared in English version and it was translated into local language (Bengali version) so that the respondents could easily understand all the questions. Before the start of the interview all the questions were described properly to the respondents by the interviewers as majority of the study participants were illiterate. An inelastic steel Mid Upper Arm Circumference (MUAC) tape was used to measure the MUAC of every participant. Data were collected regularly until the targeted sample size was obtained.

### 2.5 Variables

The socio-demographic features (such as age, sex, educational level, occupation, marital status, monthly family income level, family type), morbidity related characteristics (visual impairment, hearing problem, musculoskeletal pain, bedsore, food allergy, sense of thirst, dental prosthesis, diabetes mellitus, hypertension, cardiovascular disease), physical status related characteristics (neuropsychological status, mobility state, digestive problem, loss of weight), anthropometric measurements (height, weight, body mass index, mid-upper arm circumference), mini nutritional assessment score (MNA), simplified nutritional appetite questionnaire (SNAQ) and dietary history and habits of elderly (milk intake, legumes, and egg, meat and fish, fruits and vegetables, meals, fluid intake, drug intake) were assessed to investigate the research objective. As majority of study participants lived in joint family that's why study data were collected from the care givers (son, daughter and wife) of older people who had dementia. During appetite measurements of patients with mild dementia, opinions of the patients were prioritized over the opinions of their caregivers, but for patients with severe dementia opinions of the caregivers, were prioritized over the patient's own estimation.

### 2.6 Dietary and anthropometric measurements

Dietary intake was assessed by the diet history method [23]. This method was used for the analysis of food intake habits by the participants. The participants were asked about their food consumption habits throughout the day in different periods. The food consumption pattern was obtained by asking questions on the frequency of daily intake of certain food groups, and fluids such as legumes and egg, meat and fish, fruits and vegetables, milk and other fluids. Participants were also asked about their medication. The questionnaire included an estimate of the daily consumption (yes or no) of milk, legumes and egg, meat and fish, fruits and vegetables, drugs, as well as the approximate number of meals (2 meals or 3 meals) per day and a total cup of fluid intake (<3 cups, 3–5 cups, and >5 cups) per day. And the dietary intake was analyzed by sex and age.

Nutritional status was assessed using anthropometric measures including weight and height to calculate body mass index (BMI). The anthropometric measurements and body composition estimates were done in the morning. The weight and height of the participants were measured at their homes. Weight was measured twice to the closest 0.1 kg with light clothing on and without shoes by digital scale placed on a flat surface. The average of the two measurements was used in the analysis. Stadiometer was used to measure the height of the respondents without shoes and height (to the nearest 0.1 cm) was measured twice for each respondent. The average of the measurement was used in the analysis. The weight and height of each respondent were measured on their empty stomach and the respondents did not perform any stretching exercises before giving the measurements. BMI was calculated as weight (kg)/height (m^2^). Here in this study, Asian BMI cut-offs were used to define the nutritional status of the study participants. So based on Asian BMI cut-offs, the nutritional status was defined as underweight (<18.5 kgm^−2^), normal (18.5–22.9 kgm^−2^), overweight (23–24.9 kgm^−2^), pre-obese (25–29.9 kgm^−2^), and obese (≥30 kgm^−2^) [24]. For participants who were chair or bedridden as well as had curved spines, their height, and weight could not be measured through the height and weight scale. So their knee height (KH) was used to calculate their actual height. The knee height was measured on the left leg of each participant and a simple measuring tape was used to estimate the knee height. The knee height measurement was taken twice for each participant and the average of the measurement was used to measure the height of the chair or bedridden as well as curved spine patients. The following equations [2, 3] were used to measure the height of the immobilized respondents [25]:

(2) Height (cm) = [2.03 × KH (cm)] – [0.04 × Age (years)] + 64.19 (men)

(3) Height (cm) = [1.83 × KH (cm)] – [0.24 × Age (years)] + 84.88 (women)

To measure the weight of immobilized respondents the following anthropometric measures were collected twice from each respondent and the average of each anthropometric measure was used to calculate the weight of the immobilized participants. Calf circumference (CC), knee height (KH), arm circumference (AC) were measured by using an inelastic steel measuring tape and subscapular skinfold thickness (SST) was measured through a Lange skinfold caliper (Beta Technology Inc, USA). After estimating these anthropometric measures the following equations [4, 5] were used to estimate the weight of immobilized respondents [26]:

(4) Weight (kg) = [0.98 × CC (cm)] + [1.16 × KH (cm)] + [1.73 × AC (cm)] + [0.37 × SST (mm)] – 81.69 (men)

(5) Weight (kg) = [1.27 × CC (cm)] + [0.87 × KH (cm)] + [0.98 × AC (cm)] + [0.4 × SST (mm)] – 62.35 (women)

MUAC tape was used to measure the mid-upper arm circumference of the participants. To measure the MUAC of each respondents the MUAC tape which was provided by ACF (Action Contre la Faim) Bangladesh was used in this study. The participants were asked to bend their non-dominant arm at a right angle with the palm up. The distance between the acromial surface of the scapula and the olecranon process of the elbow on the back of the arm was measured. The midpoint of that distance was pointed with a pen and the participants were asked to let the arm hang loosely by his/her side. After that, the MUAC tape was positioned at the midpoint on the upper arm and tightened snugly. The measurement was recorded in centimeters (cm) to the nearest 0.05 cm. MUAC was measured twice for each participant and their average value was recorded as the final MUAC. The MUAC measurement procedure described in the pocket guide to nutrition and diet therapy, 1993, and a pocket guide to clinical nutrition was followed in this study [27, 28].

### 2.7 Nutritional assessment tools

The Mini Nutritional Assessment (MNA) tool was used to identify the nutritional status of the study population. This tool is recognized as a gold standard in geriatric nutrition [29]. This tool has a sensitivity of 96% and a specificity of 98% [30]. MNA has 18 items such as anthropometric measurements, dietary questionnaires, global health and social assessment, and subjective assessment of health and nutrition [31]. The total MNA score ranges from 0 to 30. The MNA score ≥24 recognizes the elderly participants as good nutritional status, the score between 17 to 23.5 means at risk of malnutrition, and MNA score less than 17 recognizes protein caloric malnutrition [32].

SNAQ is another nutritional assessment tool that was used in this study. The SNAQ tool was used in this study because it is established as a more reliable tool for subjects 60 years and above age. SNAQ tool consists of appetite, hunger, and sensory perception questionnaire [33]. SNAQ contains 4 questions and these questions were asked to the study participants during the interview. Each question consists of five options such as A, B, C, D, and E. Answers were scored based on the following numerical scale: A = 1, B = 2, C = 3, D = 4, and E = 5. The total SNAQ score was calculated by adding the scores for each question. The range of the total SNAQ score is 4–20. The SNAQ score <14 means a significant risk of weight loss >5% within 6 months with a sensitivity of 81.5% and a specificity of 76.4% [34].

### 2.8 Ethical approval and consent to participate

The Ethical Review Committee, Faculty of Biological Science and Technology, Jashore University of Science and Technology Jashore, Bangladesh gave the ethical approval to conduct this study. The study participants were reassured by the researchers that their names would not be recorded and mentioned in this study. An opportunity was given to the respondents to ask any question regarding this study and they could leave or stop the interview at any moment they wished. Written informed consent of each of the participants was obtained before data collection by explaining the purpose and methods of the study, risks, and benefits of participation in the study.

### 2.9 Data quality control

A training regarding the study objective and data collection tools was undertaken during the study. Information about essential technical skills required to collect anthropometric measurements, diet history, simplified nutritional appetite, and MNA also comprised part of the training. During this study, proper checking and supervision of the data for consistency and completeness were carried out.

### 2.10 Statistical analysis

The collected data were analyzed by using SPSS (Statistical Package for the Social Sciences) version 16 (SPSS Inc., Chicago, USA). Proper parametric and nonparametric analysis was performed. Parametric analysis was performed for continuous variables and nonparametric analysis was performed for categorical variables. Continuous variables were presented as mean ± standard deviation (SD) where applicable and categorical variables were expressed as the number and the percentage. Chi-square test and odds ratio (OR) with 95% confidence intervals (CIs) were also performed. P < 0.05 was considered statistically significant.

## 3 Results

### 3.1 Study population

The present study involved 320 participants. Demographic characteristics of the study participants are presented in Table 1. Most of the participants were male (53.4%) and their mean (SD) age was approximately 68 (6.5) years. In addition, the majority of the study participants lived in a joint family which was almost 61% and 57% for males and females respectively. Regarding educational background most of the respondents were illiterate and the percentage of illiteracy among women (65.8%) was significantly higher than that among men (57.9%). In addition, a high proportion of the study participants were farmers where 62.0% and 66.4% of male and female respondents respectively had monthly family income between 1 to 5000 taka.

**Table 1:** Socio-demographic and Socio-economic profile of the respondents (N = 320).

Characteristics		Male	Characteristics		Female
**Age in year**			**Age in year**		
Mean ± SD		67.25 ± 6.5	Mean ± SD		67.32 ± 7.7
	N	%		N	%
**Male sex**	171	53.4	**Female sex**	149	46.6
**Marital status**			**Marital status**		
Married	127	74.3	Married	93	62.4
Widow	44	25.7	Widow	56	37.6
**Family type**			**Family type**		
Nuclear	67	39.2	Nuclear	64	43.0
Joint	104	60.8	Joint	85	57.0
**Educational level**			**Educational level**		
Illiterate	99	57.9	Illiterate	98	65.8
Primary	45	26.3	Primary	41	27.5
Secondary	20	11.7	Secondary	7	4.7
College/University	7	4.1	College/University	3	2.0
**Employment**			**Employment**		
Business	14	8.2	Business	17	11.4
Salary	3	1.8	Salary	3	2.0
Farming	105	61.4	Farming	84	56.4
Labour	20	11.7	Labour	21	14.1
No cash income	26	15.2	No cash income	22	14.8
No answer	3	1.8	No answer	2	1.3
**Family income monthly**			**Family income monthly**		
1-5000 taka	106	62.0	1-5000 taka	99	66.4
5001-15000 taka	37	21.6	5001-15000 taka	23	15.4
>15000 taka	2	1.2	>15000 taka	5	3.4
Don't know	26	15.2	Don't know	22	14.8

SD = Standard deviation.

### 3.2 The dietary habit of the respondents

Table 2 summarizes the participant's dietary habits according to their sex and age group. The participants were categorized according to their sex (male or female) and age group in the year (60–75, 76–85, and >85). Almost 75% male and 70% female respondents ate legumes and eggs daily as well as 97.1% male and 99.3% female respondents ate fruits and vegetables daily. However, the intake of milk, meat, and fish was significantly lower among both males and females. The estimated per day drug intake for participants (n = 290) aged between 60-75 years was 58.3% and approximately 94% of respondents who had an age between 60-75 years took three meals a day. The fluid intake pattern was significantly lower with the increasing age of the respondents.

**Table 2:** Dietary habit of the participants (N = 320).

Eating habit/day	Gender (N = 320)		Age group (Year) (N = 320)		
	Male (n = 171)	Female (n = 149)	60-75 (n = 290)	76-85 (n = 20)	>85 (n = 10)
**Milk intake**					
Yes	48 (28.1%)	42 (28.2%)	83 (28.6%)	4 (20.0%)	3 (30%)
NO	123 (71.9%)	107 (71.8%)	207 (71.4%)	16 (80.0%)	7 (70%)
**Legumes and Egg**					
Yes	127 (74.3%)	103 (69.1%)	213 (73.4%)	10 (50.0%)	7 (70.0%)
No	44 (25.7%)	46 (30.9%)	77 (26.6%)	10 (50.0%)	3 (30.0%)
**Meat and Fish**					
Yes	67 (39.2%)	38 (25.5%)	96 (33.1%)	5 (25.0%)	4 (40.0%)
No	104 (60.8%)	111 (74.5%)	194 (66.9%)	15 (75.0%)	6 (60.0%)
**Fruits and Vegetables**					
Yes	166 (97.1%)	148 (99.3%)	287 (99.0%)	18 (90.0%)	9 (90.0%)
No	5 (2.9%)	1 (.7%)	3 (1.0%)	2 (10.0%)	1 (10.0%)
**Drug intake**					
Yes	110 (64.3%)	82 (55.0%)	169 (58.3%)	15 (75.0%)	8 (80.0%)
No	61 (35.7%)	67 (45.0%)	121 (41.7%)	5 (25.0%)	2 (20.0%)
**Meals**					
2 meals	18 (10.5%)	4 (2.7%)	19 (6.6%)	2 (10.0%)	1 (10.0%)
3 meals	153 (89.5%)	145 (97.3%)	271 (93.4%)	18 (90.0%)	9 (90.0%)
**Fluid intake**					
<3 cups	11 (6.5%)	16 (10.7%)	13 (4.5%)	9 (45.0%)	5 (50.0%)
3-5 cups	77 (45.0%)	53 (35.6%)	123 (42.4%)	5 (25.0%)	2 (20.0%)
>5 cups	83 (48.5%)	80 (53.7%)	154 (53.1%)	6 (30.0%)	3 (30.0%)

### 3.3 Nutritional status among participants of different age groups

According to BMI classification (Asian cut-off), only 2 (20.0%) respondents belonged to the normal weight that had age above 85 years. It was noticed that with advancing age the percentage of underweight was also increased such as for 60–75 years old age group the underweight percentage was 30.0% where for 76 to 85 and >85 years old age group the underweight percentage was 45.0% and 60.0% respectively. The mean (SD) value of BMI and MUAC for different age group participants are summarized in Table 3.

**Table 3:** Nutritional status among participants of different age groups (N = 320).

Variables	Age Group		
	60-75 (n = 290)	76-85 (n = 20)	>85 (n = 10)
**BMI (Kgm**^**−2**^**) Asian**			
Mean ± SD	21.028 ± 3.766	19.546 ± 2.717	18.690 ± 3.189
Underweight	87 (30.0%)	9 (45.0%)	6 (60.0%)
Normal	138 (47.6%)	7 (35.0%)	2 (20.0%)
Overweight	35 (12.1%)	3 (15.0%)	1 (10.0%)
Pre obese	25 (8.6%)	1 (5.0%)	1 (10.0%)
Obese	5 (1.7%)	0 (0.0%)	0 (0.0%)
**MNA score**			
Malnourished	84 (29.0%)	8 (40.0%)	5 (50.0%)
At risk of malnutrition	144 (49.7%)	9 (45.0%)	2 (20.0%)
Normal nutritional status	62 (21.4%)	3 (15.0%)	3 (30.0%)
**MUAC in cm**			
Mean ± SD	24.98 ± 2.31	23.15 ± 2.18	21.90 ± 1.50
**SNAQ score**			
<14	154 (53.1%)	12 (60.0%)	6 (60.0%)
≥14	136 (46.9%)	8 (40.0%)	4 (40.0%)
**Self-view (NS)**			
Malnourished	42 (14.5%)	3 (15.0%)	1 (10.0%)
Uncertain of NS	230 (79.3%)	15 (75.0%)	9 (90.0%)
No nutritional problem	18 (6.2%)	2 (10.0%)	0 (0.0%)

BMI = Body Mass Index, SD = Standard Deviation, MNA = Mini Nutritional Assessment, NS = Nutritional Status, SNAQ = Simplified Nutritional Appetite Questionnaire, Kg = Kilogram, m = Meter, cm = Centimetre.

In addition to the MNA score, the prevalence of malnutrition (n = 97) was increased with increasing age. About 50% of malnourished (as MNA score) elderly respondents had age above 85 years where only 29% and 40% malnourished (as MNA score) participants had an age between 60 to 75 and 76–85 years. According to SNAQ, the proportion of elderly respondents who had SNAQ score below 14 was 172/320. The detail of nutritional status has been demonstrated in Table 3.

### 3.4 Correlation among health complications and nutritional status according to MNA score

A significant majority of older people suffered from multiple health complications and the detail of health complications are summarized in Table 4. The majority of participants had severe and mild dementia which was significantly associated with nutritional status (P = .000, Chi square = 49.804). In addition, the percentage of people who had severe loss of appetite and moderate loss of appetite with malnutrition was 57.9% and 41.2% respectively and these changes were predominantly significant with nutritional status (P = .000, Chi square = 76.034). A total of 111 individuals lost their weight >3 kg during the last months and this weight loss tendency was significantly associated with the nutritional status of older people (P = .000, Chi square = 1.399).

**Table 4:** Correlation among health complications and nutritional status according to MNA score.

Variables	Nutritional status (MNA score)			χ2	P
	Malnourished (n = 97) n (% row wise)	At risk of malnutrition (n = 155) n (% row wise)	Well nourished (n = 68) n (% row wise)		
**Neuropsychological problem**					
Severe dementia	27 (50.0%)	24 (44.4%)	3 (5.6%)		
Mild dementia	49 (46.2%)	35 (33.0%)	22 (20.8%)	49.804	.000
No problem	21 (13.1%)	96 (60.0%)	43 (26.9%)		
**Mobility**					
Bed/chair bound	4 (66.7%)	2 (33.3%)	0 (0%)		
Able to get out of bed/chair but unable to go out	17 (53.1%)	14 (43.8%)	1 (3.1%)	16.689	.002
Able to go out	76 (27.0%)	139 (49.3%)	67 (23.8%)		
**Digestive, chewing, and swallowing problem**					
Severe loss of appetite	22 (57.9%)	16 (42.1%)	0 (0%)		
Moderate loss of appetite	63 (41.2%)	75 (49.0%)	15 (9.8%)	76.034	.000
No loss of appetite	12 (9.3%)	64 (49.6%)	53 (41.1%)		
**Loss of weight during last months**					
>3 kg	62 (55.9%)	48 (43.2%)	1 (0.9%)		
Don't know	22 (40.7%)	31 (57.4%)	1 (1.9%)	1.399E2	.000
1–3 kg	5 (9.6%)	36 (69.2%)	11 (21.2%)		
No weight loss	8 (7.8%)	40 (38.8%)	55 (53.4%)		

Statistically significant at p < 0.05, MNA = Mini Nutritional Assessment, χ2 = Chi square.

### 3.5 Morbidity characteristics of the participants

Among 320 subjects 181 (56.6%) had visual impairment. Overall, visual impairment was a little bit higher among males than in females (OR = 1.24, 95% CI = 0.799–1.939). Males had a higher proportion of hearing problems, 66.7% (114/171) compared with females, 55.7% (83/149), which was statistically significant. A total of 72.5% (232/320) elderly had dental prostheses and the dental prosthesis was slightly higher among males than in females (OR = 1.14, 95% CI = 0.695–1.857). In addition, diabetes mellitus, hypertension, and cardiovascular disease were significantly present among the respondents and the detail about morbidity characteristics can be seen in Table 5.

**Table 5:** Morbidity characteristics of the participants (N = 320).

Variables	Present (%)	Absent (%)	Total (%)	OR (95% CI)
**Visual impairment**				
Males	101 (59.1%)	70 (40.9%)	171 (100%)	
Females	80 (53.7%)	69 (46.3%)	149 (100%)	
Total	181 (56.6%)	139 (43.4%)	320 (100%)	1.24 (.799–1.939)
**Hearing problem**				
Males	114 (66.7%)	57 (33.3%)	171 (100%)	
Females	83 (55.7%)	66 (44.3%)	149 (100%)	
Total	197 (61.6%)	123 (38.4%)	320 (100%)	1.59 (1.011–2.503)
**Musculoskeletal pain**				
Females	60 (40.3%)	89 (59.7%)	149 (100%)	
Males	66 (38.6%)	105 (61.4%)	171 (100%)	
Total	126 (39.4%)	194 (60.6%)	320 (100%)	1.073 (.684–1.681)
**Bedsore**				
Females	20 (13.4%)	129 (86.6%)	149 (100%)	
Males	13 (7.6%)	158 (92.4%)	171 (100%)	
Total	33 (10.3%)	287 (89.7%)	320 (100%)	1.884 (.903–3.934)
**Food allergy**				
Males	50 (29.2%)	121 (70.8%)	171 (100%)	
Females	43 (28.9%)	106 (71.1%)	149 (100%)	
Total	93 (29.1%)	227 (70.9%)	320 (100%)	1.02 (.628–1.653)
**Sense of thirst**				
Females	70 (47.0%)	79 (53.0%)	149 (100%)	
Males	70 (40.9%)	101 (59.1%)	171 (100%)	
Total	140 (43.8%)	180 (56.2%)	320 (100%)	1.278 (.821–1.991)
**Dental problem**				
Males	126 (73.7%)	45 (26.3%)	171 (100%)	
Females	106 (71.1%)	43 (28.9%)	149 (100%)	
Total	232 (72.5%)	88 (27.5%)	320 (100%)	1.14 (.695–1.857)
**Diabetes Mellitus**				
Males	101 (59.1%)	70 (40.9%)	171 (100%)	
Females	80 (53.7%)	69 (46.3%)	149 (100%)	
Total	181 (56.6%)	139 (43.4%)	320 (100%)	1.24 (.799–1.939)
**Hypertension**				
Males	112 (65.5%)	59 (34.5%)	171 (100%)	
Females	92 (61.7%)	57 (38.3%)	149 (100%)	
Total	204 (63.8%)	116 (36.2%)	320 (100%)	1.18 (.745–1.857)
**CVD**				
Males	113 (66.1%)	58 (33.9%)	171 (100%)	
Females	94 (63.1%)	55 (36.9%)	149 (100%)	
Total	207 (64.7%)	113 (35.3%)	320 (100%)	1.14 (.720–1.804)

CVD = Cardio Vascular Disease, OR = odd ratio, CI = confidence interval.

## 4 Discussion

It has been ensured through the present cross-sectional study that malnutrition and age-related health complications remain a common problem among older people living in rural areas of Bangladesh. The mean age of male participants was 67.25 years whereas, the mean age of female participants was 67.32 years. This study included those people who had age ≥60 years because people with age not less than 60 years are defined as older people [5]. So the selection of older people for this study which was based on age category was very correct. According to this study 57.9% male and 65.8%, female respondents had no formal education. In addition, the rate of literacy among the male participants was higher than the female participants. A similar result was also noticed in a study conducted in Narayanganj district Bangladesh where the mean age for males was 67.69 years and for females, the mean age was 65.46 years. They also selected individuals not less than 60 years where the male literacy was three times higher than the female literacy [6]. However, this study result shows the opposite result with a study in Taiwan where the majority of their older respondents were educated [35]. From this study, it was found that 74.3% male and 62.4% female respondents were married as well as 60.8% male and 57% female respondents lived in a joint family. So the majority of this study respondents were married and lived in a joint family. A vast of this study participants belonged to a family with a monthly family income between 1 to 5000 taka and it was an indication of low socioeconomic condition. This result is very much similar to another study conducted in three different districts (Sylhet, Mymensingh, and Noakhali) of Bangladesh where 85% (majority) elderly were married and 53.7% (majority) elderly lived in a joint family [36]. Another study in Bangladesh shows that it is a tradition of Bangladesh that older people are very much comfortable to live in a joint family with their children and a monthly family income of the older people below 5000 takas has shown by their study [37]. Although few respondents have obtained formal education and few have good economic conditions, the majority of participants in the current study reported no formal education and poor economic condition. So this study assumed that the nutritional status of the study participants may be affected by these socio-demographic and socio-economic factors. This is especially concerning because some previous studies reported a strong correlation among age, gender, marital status, educational level, household income, and nutritional status [38, 39, 40, 41].

Within the study sample majority of the respondents ate fruits, vegetables, legumes, and eggs daily which was because most of the respondents belonged to the agricultural family as well as all of them lived in rural areas where home gardening of different fruits and vegetables was a common scenario. On the other hand, approximately 61% male and 75% female respondents did not eat meat and fish daily. However a significant portion of this study participants ate meat and fish daily because this study was conducted in rural areas of Jashore where the meat and fish production was higher by the villagers in comparison with other areas of Bangladesh. During the survey it was noticed that some households had domestic animals such as chickens, goats, cows etc and there was also village poultry farms which might be the major potential source of meat for the villagers. In addition, it was also noticed that each village had a lot of fish ponds where the villagers usually harvested a significant amount of fishes to meet their daily needs and they also supplied it to the local markets to earn some livelihoods. In addition, almost 72% of male and female respondents did not consume milk daily. Milk, meat, and fish are great sources of protein and fat which the respondents lacked off. On the other hand majority of this study respondents were illiterate and a vast of them belonged to a poor monthly income family. So it was very difficult for this study respondents to buy these protein and fat-rich foods because in Bangladesh's perspective these foods are very expensive. The lacking of these protein-rich foods contributed much to the nutritional status of the older people. A study in Bangladesh among rural elderly reported that only 10.2% male and 13.5% female respondents had protein-rich food more than five days a week. Like this study, the majority of their males and females had no opportunity to take protein-rich foods daily [6]. Another study in Ghana reported that fish was consumed daily by the elderly apart from fruits and vegetables and their study showed a little bit different result from this study [42]. This may be happened because of the difference in the characteristics and profile of the study population. Although few respondents have consumed protein-rich foods daily, the majority of participants in the current study did not have the opportunity to take protein-rich foods daily. As a result, a large portion of older people was very much vulnerable to malnutrition. It is especially concerning because a study proved that poor economic status, inappropriate dietary intake, and social deprivation contributed to the vulnerability of malnutrition among older people [43].

Through the use of anthropometric measurements, a study in rural Malawi mentioned that undernutrition among its elder people was a significant problem [44]. Based on BMI and mid-upper arm circumference the prevalence of malnutrition in central Uganda among 60–90 years old, aged people was 33% and 52% respectively [45]. However, in this study a total of 102 (31.9%) respondents had underweight and 39 (12.2%) respondents had overweight according to the Asian BMI cut-off. On the other hand, a study in eastern Poland among rural older people aged 60 years and above reported that 2.8% of their participants were underweight, 37.6% were overweight and 33.8% were obese. They used the WHO BMI cut-off to measure the nutritional status of rural elderly in eastern Poland [46]. In addition, a study among 60 years old people who came to Bangabandhu Sheikh Mujib Medical University, Bangladesh mentioned that 24%, 32%, 32%, and 12% of their respondents were underweight, normal, overweight, and obese respectively [4]. This study results are comparable with the study in Cuba, China, and Italy where the prevalence of undernutrition among the elderly according to their BMI cut-off was 33%, almost 59%, and almost 12% respectively [47, 48, 49]. As BMI measurement is difficult due to deformity or disability among the elderly so BMI is not always an appropriate tool to measure the nutritional status among the elderly [50]. That is the main reason besides BMI, other tools such as MUAC, MNA, and SNAQ were used in this study to assess the nutritional status of the subjects. Within this sample of respondents who had an age between 60-75 years, their mean (SD) value for MUAC was 24.98 (2.31) and there was a significant decrease in the mean value of MUAC with the advancing age of the respondents. This is a matter of great concern because in a study to detect undernutrition among Bangladeshi adults a MUAC of <25.1cm was established as optimal cut-off [51]. However, a study among five African countries, India, China, and Papua New Guinea established a MUAC of 23 cm as a cut-off for identifying nutritional status [52]. To identify undernutrition various studies in India among different ethnic groups used MUAC of <23cm as cut off [53, 54]. According to a study in Taiwan, a MUAC of <23.5 cm was significantly associated with MNA (Mini Nutritional Assessment) predicted malnutrition risk among the elderly [55]. So this study, also used the MNA tool besides MUAC measurement. A previous study which was conducted in Bangladesh through the use of an MNA questionnaire mentioned a high prevalence of malnutrition and at risk of malnutrition among the rural elderly (26% and 62% respectively) [56]. Compared with the results from a study conducted by using the MNA tool among rural elderly of western Rajasthan [57] this study also found a high prevalence of malnutrition and at risk of malnutrition among the study population (30.3% and 48.4% respectively according to the MNA score). However, a large study in Spain demonstrated a much fewer rate of malnutrition and at risk of malnutrition (4.3% and 25.4%, respectively) than this study [58]. From this study, it was found that the rate of at risk of malnutrition was higher than the rate of malnutrition among the study population. This finding is in line with other studies conducted among community-dwelling elderly from India and other parts of the world [57, 58, 59, 60]. In addition, a study among non-institutionalized elderly persons in the USA and Europe reported that no elderly were suffered from malnutrition but the prevalence of nutritional risk was between 18% and 41% [60, 61, 62]. In this study, a lower MNA score was associated with the older age of the study population. Various previous studies showed the same result as this study [56, 58] but some other studies mentioned no significant effect of age on the nutritional status of the elderly [63, 64]. So from this study, it is evident that more focus should be given on the nutritional status of the elderly as their age increases. Some studies reported that Bangladesh is experiencing the phenomenon of the ‘double burden of malnutrition’, namely the coexistence of overweight and undernutrition [65]. However, this did not find in that study because this study was conducted in rural areas where majority of the respondents were illiterate and belonged to poor socioeconomic condition. For this reason the severity of undernutrition was higher than over weight among the study respondents. Though a huge portion of this study participants belonged to the group of malnutrition or at risk of malnutrition however majority of the study respondents ate 3 meals per day. One possible explanation about that majority of this study respondents followed an inappropriate dietary pattern which's why a large portion of them was malnourished. This result was slightly different from another study report which was conducted in Bangladesh where the majority of their elderly consumed only 2 meals per day and most of their respondents were in the group of malnutrition or at risk of malnutrition [56]. Within this study sample, about 172 (53.8%) participants had SNAQ values below 14 and that expressed a significant risk of weight loss by the study population. It is especially concerning because weight loss is recognized as a vital index of nutritional risk in older adults as well as is an indicator of morbidity and mortality among the elderly [66, 67]. So this is the main reason that a total of 111 (34.7%) respondents lost their weight above 3 kg during the last months and approximately 56% of them were malnourished. This study found a statistically significant correlation among various health complications (neuropsychological problem, mobility complications, digestive problem, and loss of weight during last month's) with nutritional status according to MNA score. In this study, 106 participants had mild dementia and among them 46.2% of respondents were malnourished. However half of the study participants had no neuropsychological problem. This study result was very much similar to a study in India where the majority of their respondents had no neuropsychiatric problem and only 20% of their respondents had mild dementia [15]. It was noticed that within this study sample a total of 38 and 153 respondents had a severe and moderate loss of appetite respectively. Almost 58% of respondents who had severe loss of appetite were malnourished as well as approximately 42% of respondents who had a moderate loss of appetite were also malnourished in this study due to chewing, digestive and swelling problems. This is a matter of great concern because previous studies revealed that chewing problems and difficulty to eat full meals contributed to malnutrition among the community-dwelling elderly [57, 63]. It was noticed that a total of 282 (88.1%) participants of this study were able to move or go out but 27.0% and 49.3% respondents among them were in the group of malnutrition and at risk of malnutrition respectively which was a matter of concern for this study population. This result was very much similar to another study conducted in India where 94% of their respondents were able to go out [15]. The most common morbidities suffered by the elderly in this study were visual impairment (56.6%), hearing problem (61.6%), dental problem (72.5%), diabetes mellitus (56.6%), hypertension (63.8%), and cardiovascular disease (64.7%). A majority of this study respondents had several morbidities which was the reason a vast of the study participants took three prescription drugs per day. On the other hand, it was noticed from this study that with advancing age there was a decrease in the rate of fluid intake per day by the respondents. One possible explanation is that 56.2% of respondents had no sense of thirst which leads to reduce fluid consumption by the respondents. In western countries, the most common morbidities among the elderly are mental disorders, obesity, and cancer which were not seen in this study [68]. However, the morbidity characteristics of this study participants were very much comparable with the morbidity incidence of different Asian countries like India, Malaysia, and South Korea [69, 70, 71]. According to a study among the elderly in urban areas of Dhaka, Bangladesh reported that the most common health problems among their participants were vision problems followed by hearing problems and cardiovascular disease which were very much similar to this study result [5]. Like this study, visual impairment and hearing problems were significantly higher in males than females in a study conducted in India [72]. Compared to this study only a few respondents (23.9%) had dental problems in an Indian study [73]. In addition, a statistical survey by American Heart Association among older Americans reported that 62.0 men and 67.8% of women aged between 65 to 74 years had high blood pressure. The same statistical survey reported that 69.1% male and 67.9% female who aged between 60-79 years had cardiovascular disease [74]. As there is a high prevalence of diabetes and hypertension in this study so it is a matter of great concern for the study population because diabetes and hypertension are recognized as the vital risk factors to develop CVDs [75]. Since several morbidities of the study participants such as diabetes mellitus, hypertension, CVDs, dental problem could affect their food intake pattern so it is assumed that there might be a relationship between the morbidity and nutritional status of the older people.

### 4.1 Strengths and limitations

To the best of knowledge, the main strength of this study is that this is the first study carried out in Bangladesh, using large sample size and a comprehensive nutritional assessment including anthropometric measurements and dietary habit assessment, to evaluate nutritional status and health complications of older adults. In addition, this is the only study in Bangladesh that has used BMI, SNAQ questionnaire, MUAC, diet history method, and MNA tool together to estimate the nutritional status and health complications of the elderly. The main limitation of this study is that it could not conduct clinical examination as well as biochemical analysis to assess the nutrient deficiency in older adults. The cross-sectional design of the study and the generalizability of the results are another potential limitations of this study. In addition, another limitation of this study is that the investigators could not observe the quality and quantity of the food consumed by the study population.

## 5 Conclusions

The present study has highlighted that inadequate nutritional status including underweight, at risk of malnutrition, poor intake of protein-rich foods, loss of weight above 3 kg during last months, decreased fluid intake, lower MNA score, lower SNAQ score and lower MUAC value were prevalent among older adults. It has also mentioned that visual impairment, hearing problems, dental problems, diabetes mellitus, hypertension, cardiovascular disease, absence of thirst sensation, digestive problem, neuropsychological problem, and mobility problems were ubiquitous in the elderly. Therefore, the results of this study shed light on the current nutritional status as well as health complications in rural elderly of Bangladesh to initiate effective interventions to address this issue by policymakers, public health researchers, and the government of Bangladesh.

## Declarations

### Author contribution statement

Arafat Hassan Razon: Performed the experiments; Analyzed and interpreted the data; Contributed reagents, materials, analysis tools or data; Wrote the paper.

Md.Imamul Haque, Md.Foyaj Ahmed: Performed the experiments.

Tanvir Ahmad: Conceived and designed the experiments; Analyzed and interpreted the data; Contributed reagents, materials, analysis tools or data.

### Funding statement

This research did not receive any specific grant from funding agencies in the public, commercial, or not-for-profit sectors.

### Data availability statement

Data will be made available on request.

### Declaration of interests statement

The authors declare no conflict of interest.

### Additional information

No additional information is available for this paper.
